# Epigenetic regulation of breast ductal carcinoma in situ by miR‐217 through DNMT1 and Hedgehog‐GLI pathway

**DOI:** 10.1002/ccs3.70030

**Published:** 2025-09-03

**Authors:** Zixin Wang, Liangping Wu, Shuhui Lai, Sixuan Guo, Changqin Pu, Linyi Zhang, Xiaoling Li

**Affiliations:** ^1^ Department of Metabolic Surgery Jinshazhou Hospital of Guangzhou University of Chinese Medicine Guangzhou China; ^2^ The First Clinical Medical College Nanchang University Nanchang China; ^3^ The Second Clinical College Medical College of Nanchang University Nanchang China; ^4^ Queen Mary of Nanchang University Nanchang China; ^5^ School of Ophthalmology and Optometry Nanchang University Nanchang Jiangxi China; ^6^ Department of Anatomy School of Basic Medical Sciences, Qiqihar Medical University Qiqihar China

**Keywords:** breast ductal carcinoma in situ, DNA methyltransferase 1, Hedgehog‐GLI pathway, miR‐217, teashirt zinc finger homeobox 2

## Abstract

Ductal carcinoma in situ (DCIS) is a noninvasive precursor of breast cancer with a high potential for progression. Aberrant DNA methylation plays a pivotal role in early tumorigenesis, yet the regulatory mechanisms remain incompletely defined. Integrated bioinformatic analysis of methylation and transcriptomic datasets identified miR‐217 as a candidate regulator of DNA methyltransferase 1 (DNMT1). Functional validation was conducted through RT‐qPCR, dual‐luciferase reporter assays, methylation‐specific PCR, chromatin immunoprecipitation, and phenotypic assays in ZR‐75‐1 cells. An in vivo xenograft model using nude mice was employed to verify the regulatory axis. Expression of miR‐217 was significantly reduced in DCIS tissues and inversely correlated with DNMT1 levels. Direct binding between miR‐217 and the 3′ untranslated region of DNMT1 was confirmed. Overexpression of miR‐217 suppressed DNMT1, resulting in hypomethylation of the teashirt zinc finger homeobox 2 (TSHZ2) promoter and restoration of TSHZ2 expression. Elevated TSHZ2 inhibited Hedgehog‐GLI signaling, thereby reducing cell proliferation, migration, invasion, and tumorigenic potential. Reintroduction of DNMT1 or activation of Hedgehog‐GLI signaling reversed these effects. In vivo, miR‐217 overexpression suppressed tumor growth, downregulated DNMT1 and GLI1, and increased apoptosis. The miR‐217/DNMT1/TSHZ2/Hedgehog‐GLI signaling axis modulates DCIS progression by epigenetically reprogramming oncogenic pathways. Targeting this axis may offer a promising strategy for DCIS treatment.

## Graphical abstract

This study reveals that miR‐217 suppresses breast ductal carcinoma in situ (DCIS) by targeting DNMT1, reducing TSHZ2 promoter methylation and restoring TSHZ2 expression. TSHZ2 inhibits the Hedgehog‐GLI signaling pathway, thereby limiting tumor proliferation, migration, and invasion. Both in vitro and in vivo results underscore miR‐217's therapeutic potential in DCIS treatment.

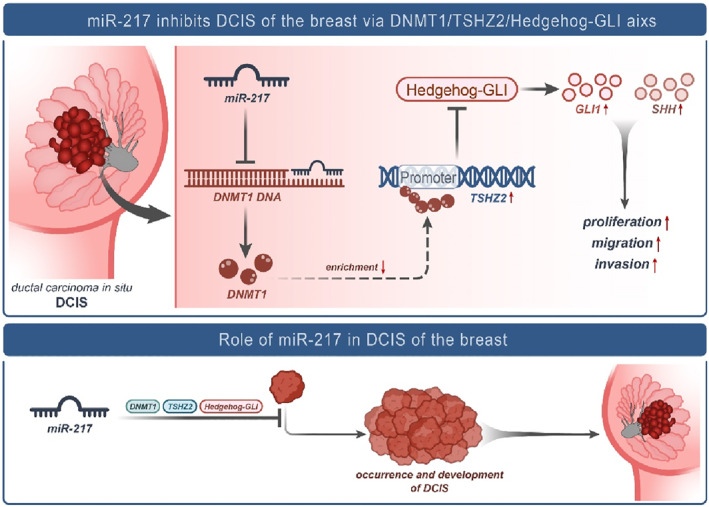

## 1 INTRODUCTION

Ductal carcinoma in situ (DCIS) is a noninvasive form of breast cancer (BC), with incidence rates increasing due to the widespread implementation of BC screening programs.^1^ Identified risk factors encompass demographic, lifestyle, and reproductive variables, including age, physical activity, height, body mass index, family history of BC, menopausal status, and parity.^2^ Despite the availability of potential treatments, including surgical excision, radiation therapy, hormonal therapy, and palliative care, most DCIS lesions remain asymptomatic, leading to frequent over‐treatment in clinical practice.^3^, ^4^ The development of predictive biomarkers is required to enhance the diagnosis and management of DCIS.

MicroRNAs (miRNAs or miRs) play a crucial role in the early prognosis and diagnosis of DCIS.^5^, ^6^ Among them, miR‐217 has been implicated in the pathogenesis of various human diseases.^7^ In BC, miR‐217 is consistently underexpressed in BC tissues and cells. Its overexpression markedly suppresses malignant cellular behavior, thereby limiting BC progression.^8^ DNA methyltransferase 1 (DNMT1) is proposed as a direct target of miR‐217, whereas inhibition of DNMT1 activity effectively suppresses tumor growth.^9^ DNMT1 is essential for maintaining DNA methylation.^10^ It is worth noting that methylation of the teashirt zinc finger homeobox 2 (TSHZ2) gene promoter has been detected in the MDA‐MB‐231 BC cell line.^11^ In triple‐negative breast cancer (TNBC), TSHZ2 mRNA expression is markedly reduced and may serve as a predictive marker for TNBC metastasis.^12^ As previously mentioned, downregulation of TSHZ2 leads to activation of glioma‐associated oncogene homolog 1 (GLI1), a downstream transcription factor in the Hedgehog signaling pathway.^13^ The Hedgehog pathway serves as a key regulator of embryonic development and stem cell dynamics and contributes to BC progression by enhancing epithelial‐mesenchymal transition, sustaining cancer stem cell populations, promoting angiogenesis and increasing cellular invasiveness.^14^ To investigate the functional relevance of this signaling cascade, patient‐derived DCIS specimens were collected and analyzed through a series of in vivo and in vitro experiments to elucidate the involvement of the miR‐217/DNMT1/Hedgehog‐GLI axis in the progression of DCIS.

## 2 MATERIALS AND METHODS

### 2.1 Ethical statement

This study was approved by the Clinical Ethics Committee of Qiqihar Medical University (Approval No. 2022‐65) and conducted in accordance with the Declaration of Helsinki. Written informed consent was obtained from all participants. All animal experiments were approved by the Animal Ethics Committee of Qiqihar Medical University (Approval No. QMU‐AECC‐2022‐83) and conducted in strict adherence to the “Guide for the Care and Use of Laboratory Animals” published by the National Institutes of Health.

### 2.2 Clinical sample collection

A total of 40 patients with DCIS were recruited from the First Clinical Medical College of Nanchang University between May 2016 and May 2017. The median age of the patients was 52 years (range 42–68 years). All cases met the DCIS criteria outlined in the American Joint Committee on Cancer Staging Manual, which specifies the presence of malignant cells confined within the breast ducts without stromal invasion. Detailed clinical records were available for all patients, and adjacent normal tissues from the same patients were selected as controls.

### 2.3 Bioinformatics analysis

Methylation sequencing data for 24 DCIS tissues and 5 normal tissues were retrieved from the Sequence Read Archive (SRA) database using reduced representation bisulfite sequencing (RRBS) (accession numbers: SRR2069903 to SRR2069931). After quality control, differentially methylated regions were identified using the R packages methylKit and eDMR. These regions were annotated, and common genes were extracted from the intersection of the methylKit and eDMR results based on methylKit‐derived annotations. DCIS‐related mRNA expression microarrays were retrieved from the Gene Expression Omnibus (GEO) database. Differential expression analysis was performed to identify significantly downregulated mRNAs in DCIS, which were subsequently intersected with genes exhibiting promoter hypermethylation. The expression of TSHZ2 in BC samples was validated using the GEPIA database. CpG islands in the TSHZ2 promoter region were predicted using MethPrime, and potential binding sites for DNMT1 on the TSHZ2 promoter were identified using the BLAST online tool. Additionally, candidate microRNAs predicted to target DNMT1 were retrieved using the microRNA Data Integration Portal (mirDIP). MicroRNA expression microarrays associated with BC were then screened from the GEO database. Differential expression analysis was conducted, and significantly downregulated microRNAs in BC samples were intersected with DNMT1‐targeting microRNAs predicted by mirDIP.

### 2.4 Cell culture, grouping, and transfection

The human DCIS cell line ZR‐75‐1 (ATCC® CRL‐1500™) was purchased from the American Type Culture Collection. Cells were resuspended in Dulbecco's Modified Eagle Medium (DMEM) containing 10% fetal bovine serum (FBS, Biowest) and 100 U/mL of penicillin and streptomycin (Gibco by Life Technologies), maintained at 37°C in 5% CO_2_, with regular medium changes. Upon reaching 80%–90% confluence, cells were passaged and used for experiments during the logarithmic growth phase. Cells were seeded in six‐well plates (4 × 10^5^ cells/well) and transfected according to the Lipofectamine 2000 protocol (11668‐019, Invitrogen) when confluence reached approximately 80%. For transfection, 2 μg of plasmid DNA was diluted in 125 μL of serum‐free Opti‐MEM medium, and 5 μL of Lipofectamine 2000 was diluted in 125 μL of serum‐free Opti‐MEM medium. The two solutions were mixed at a 1:1 ratio and incubated at room temperature for 20 min before being added to the six‐well plates. GANT 61 (Synonyms: NSC 136476) was purchased from MedChemExpress and added to the medium at a concentration of 20 μM. Cells were cultured at 37°C in 5% CO_2_, and RNA and protein were extracted at 24 and 48 h.

Cells were divided into the following experimental groups: mimic‐NC, miR‐217‐mimic, inhibitor‐NC, miR‐217‐inhibitor, mimic‐NC + oe‐NC, miR‐217‐mimic + oe‐NC, miR‐217‐mimic + oe‐DNMT1, oe‐NC, oe‐DNMT1, si‐NC, si‐DNMT1, inhibitor‐NC + oe‐NC, miR‐217‐inhibitor + oe‐NC, miR‐217‐inhibitor + oe‐DNMT1, oe‐DNMT1 + oe‐TSHZ2, siDNMT1 + siTSHZ2, oe‐TSHZ2, si‐TSHZ2, oe‐TSHZ2 + DMSO, oe‐TSHZ2 + GANT61, and si‐TSHZ2 + GANT61. Transfection reagents and plasmids were obtained from Shanghai Jima Corporation. Each experiment was repeated three times.

### 2.5 Dual‐luciferase assay

The bioinformatics website TargetScan was utilized to predict the binding sites miR‐217 on the DNMT1 3′UTR. This interaction was validated using a dual‐luciferase reporter assay. Synthetic gene fragments of DNMT1‐3′UTR (WT: GUUCCUG) were cloned into the psiCHECK‐2 luciferase vector (Promega Corporation) using SpeI and Hind III restriction sites. Mutant sequences (MUT: CAAGGAC) were designed to introduce complementary mutations in the DNMT1 wild‐type 3′ UTR. Following restriction enzyme digestion, the fragments were inserted into the psiCHECK‐2 reporter plasmid using T4 DNA ligase. Sequenced luciferase reporter plasmids WT and MUT were transfected into HEK293T cells along with negative control (mimic‐NC) and miR‐217 mimic (miR‐217‐mimic). Cells were harvested and lysed 48 h post‐transfection, and luciferase activity was measured using a luciferase assay kit (Promega Corporation) with a GloMax 20/20 luminometer (Promega Corporation).^15^ Each experiment was conducted in triplicate.

### 2.6 CCK‐8 assay

Cells in the logarithmic growth phase were seeded into 96‐well plates at a density of 2 × 10^3^ cells per well. Following transfection, according to experimental groups, the cells were incubated in a culture incubator with three replicate wells per group. At 24, 48, 72, and 96 h post‐incubation, 10 μL of CCK‐8 reagent was added to each well. The plates were further incubated for 2 h, and the optical density at 450 nm was measured using a spectrophotometer (Thermo Fisher).^16^ Each experiment was conducted in triplicate.

### 2.7 Transwell migration assay

Twenty‐four hours post‐transfection with plasmid DNA, cells were digested into a single‐cell suspension and adjusted to a concentration of 1 × 10^6^ cells/mL in a medium containing 1% FBS. In a 24‐well plate, 600 μL of complete medium containing 10% FBS was added to each lower chamber. Transwell inserts were placed into the wells, and 100 μL of the prepared cell suspension was added to each insert, ensuring the absence of air bubbles. The cells were cultured at 37°C for 24 h. Post‐incubation, the inserts were removed and nonmigratory cells and medium were wiped off with a cotton swab. The chambers were washed twice with phosphate‐buffered saline (PBS) for 2 min each. The cells were then fixed with a 3:1 methanol and acetic acid solution for 15–30 min and air‐dried. Staining was performed with 1% crystal violet solution for 20 min, followed by two additional PBS washes, each lasting 2 min. Cell migration was observed under an inverted microscope (TE2000, Nikon), and representative images were captured and analyzed. Each experiment was repeated three times.

Matrigel matrix (356234, Corning) was prepared ^17^ by incubating the reagent at 4°C overnight. Pipettes and tips were pre‐cooled at −20°C before use. The membrane was coated by diluting Matrigel in serum‐free cold DMEM on ice. A volume of 100 μL of the diluted Matrigel was added to the upper chamber of a 24‐well Transwell plate and incubated at 37°C for 4–5 h to allow solidification. The coated membrane was then gently hydrated using a serum‐free medium. Cells were digested and washed three times with medium, then resuspended at a density of 5 × 10^5^ cells/mL in DMEM containing 1% FBS. A total of 200 μL of the cell suspension was added to the upper chamber, whereas 600 μL of medium containing 5 μg/mL fibronectin was added to the lower chamber. The plate was incubated at 37°C for 20–24 h. Noninvasive cells remaining on the upper surface of the membrane were carefully removed using a cotton swab. Inserts were removed, inverted, and allowed to air dry. For staining, 500 μL of 1% crystal violet solution was added to each well, and the chamber was fully submerged and incubated at 37°C for 30 min. After staining, membranes were rinsed with PBS. Invaded cells were imaged and counted in four fields of view per membrane.

### 2.8 Monoclonal formation assay

Cells in the logarithmic growth phase were digested with EDTA +0.25% trypsin, dissociated into single cells, and resuspended in DMEM high‐glucose complete medium (Gibco) for later use. After cell counting, approximately 200 cells were seeded in 10 mL of the same medium and transferred into a 10 cm diameter cell culture dish (BD). The dish was gently rotated to ensure uniform distribution. Cells were cultured at 37°C with 5% CO_2_ for 10–14 days. Cell growth was monitored regularly, and the culture was terminated once visible colonies had formed. The supernatant was discarded, and the cells were gently washed twice with PBS, fixed with 5 mL of methanol for 15 min, and stained with Giemsa stain for 30 min. Excess stain was washed off with running water, and plates were air‐dried. Colonies containing more than 10 cells were counted either directly by visual inspection using a grid overlay or under low magnification using a microscope. The clonogenic efficiency was calculated using the formula: (number of colonies/number of seeded cells) × 100%.^18^

### 2.9 RT‐qPCR

Total RNA was extracted from tissues or cells using TRIzol (Invitrogen) or the RNeasy Mini Kit (Qiagen). For mRNA detection, complementary DNA (cDNA) was synthesized using a Reverse Transcription Kit (RR047A, Takara). For miRNA detection, cDNA was synthesized using the miRNA First Strand cDNA Synthesis (Tailing Reaction) Kit (B532451‐0020, Sangon Biotech). GAPDH and U6 were used as internal references for mRNA and miRNA quantification, respectively. Primer sequences are listed in Table S1. Reverse transcription polymerase chain reaction experiments were conducted using SYBR® Premix Ex Taq™ (Tli RNaseH Plus) (RR820A, Takara) on an ABI 7500 Real‐Time PCR System (Thermo Fisher Scientific). The PCR reaction volume was set at 25 μL. Reaction mixtures were gently flicked to mix, briefly centrifuged at 5000 r/min, and subjected to the following thermal cycling conditions: initial denaturation at 95°C for 5 min followed by 40 cycles of 95°C for 10 s, annealing at primer‐specific temperatures for 20 s, extension at 72°C for 20 s, and final elongation at 78°C for 20 s. Data were analyzed using the 2^−ΔΔCt^ method.^18^ Each experiment was repeated three times.

### 2.10 Methylation‐specific PCR (MSP)

Methylation levels of the TSHZ2 promoter region were assessed using the DNA Methylation‐Gold™ Kit (D5005, Zymo Research). For MSP amplification, two sets of primers were designed. The methylated‐specific primers were: forward 5′‐TTATAGGAGGGTTTATAGGTATCGT‐3′ and reverse 5′‐AAAAATTTAAAAATCAAATCTCGAA‐3′. The unmethylated‐specific primers were forward 5′‐TTATAGGAGGGTTTATAGGTATTGT‐3′ and reverse 5′‐AAAAATTTAAAAATCAAATCTCAAA‐3′. PCR amplification was carried out using the following conditions: initial denaturation at 95°C for 10 min followed by 35 cycles of denaturation at 95°C for 45 s, annealing at 56°C for methylation or 45°C for unmethylation for 45 s, and extension at 72°C for 45 s followed by a final extension at 72°C for 10 min. The PCR products were subjected to agarose gel electrophoresis using a 50 bp DNA ladder as the molecular marker. Gel imaging and analysis were performed using a gel documentation and analysis system.^19^, ^20^ All experiments were repeated in triplicate.

### 2.11 Western blot analysis

Total protein was extracted from tissues or cultured cells using a RIPA lysis buffer kit (R0010, Solarbio Science & Technology Co., Ltd.), and protein concentrations were determined using a BCA Protein Assay Kit (Jebio). Equal amounts of protein (40 μg per sample) were separated by 10% SDS‐PAGE and transferred onto polyvinylidene fluoride membranes (Millipore). Membranes were blocked at room temperature with 5% nonfat milk in TBST, washed three times with TBST for 5 min each, and incubated overnight at 4°C with diluted primary antibodies against β‐Tubulin (ab210797, 1:1000, rabbit, Abcam), DNMT1 (ab196927, 1:1000, rabbit, Abcam), TSHZ2 (ab140189, 1:1000, rabbit, Abcam), GLI1 (ab217326, 1:1000, rabbit, Abcam), and Sonic Hedgehog (SHH) (sc‐365112, 1:100–1:1000, mouse, Santa Cruz Biotechnology). After washing three times with TBST for 5 min each, the membranes were incubated with goat anti‐rabbit IgG secondary antibody (ab150077, 1:1000, Abcam), on a shaker at room temperature for 1 h. The membranes were washed again three times with TBST for 10 min each and visualized using an enhanced chemiluminescence detection system. Band intensities were quantified using ImageJ software (v1.8.0.345), and the ratios of the target protein to the internal reference protein were calculated.^19^ Each experiment was repeated three times.

### 2.12 Chromatin immunoprecipitation (ChIP) assay

Cells were fixed in 4% formaldehyde to achieve a final concentration of 1%, followed by cell lysis and DNA fragmentation through sonication. Rabbit anti‐DNMT1 antibody (ab13537, 1:50, Abcam) was added to the lysates to target the DNMT1 protein bound to associated promoter regions. Protein A Agarose/Salmon Sperm DNA was then introduced to bind the DNMT1 antibody‐DNMT1‐TSHZ2 promoter complex. The complexes were precipitated and washed to remove nonspecific bindings, and enriched DNMT1‐TSHZ2 promoter complexes were eluted. Following the reversal of cross‐links, enriched TSHZ2 promoter fragments were purified and amplified by PCR using primers TSHZ2‐F (5′‐GGAGGAGTTTGTTAATGTTTAG‐3′) and TSHZ2‐R (5′‐AAAATCTAAAATTCACTCACTCACAC‐3′).^21^ Each experiment was repeated three times.

### 2.13 Nude mice tumorigenicity assay

Eighteen 6‐week‐old male athymic NCr^nu/nu^ (NCr‐NU‐Foxn1^nu^) nude mice were sourced from the Experimental Animal Center of Wuhan University. The mice were housed under specific pathogen‐free conditions in a barrier facility equipped with HEPA‐filtered laminar airflow. The environment was routinely sterilized with ultraviolet light, and all cages, bedding, water, and feed were autoclaved. The room temperature was maintained at 24–26°C with a relative humidity of 40%–60%. All procedures were approved by the Clinical Experimental Ethics Committee of the institution.

miR‐217 overexpressing lentivirus Lv‐miR‐217 + oe‐NC, co‐overexpressing miR‐217, and GLI1 lentivirus Lv‐miR‐217 + oe‐GLI1 (experimental group), and control vector lentivirus Lv‐NC + oe‐NC (control group) were constructed and packaged. Following infection, ZR‐75‐1 cells (1 × 10^6^) were subcutaneously injected into each mouse (six mice per group). Tumor growth, volume, and mass were recorded daily for 30 days. At the end of the study, mice were euthanized with a CO_2_ + 50% O_2_ mixture. Tumors were excised, and tumor size and mass were measured. Expression levels of miR‐217 and related proteins within the tumor tissues were analyzed. Methylation status was assessed using MSP and ChIP assays. Tumor sections were prepared and stained with TUNEL (6432344001, Roche) to observe apoptosis.^22^

### 2.14 Statistical analysis

All data were analyzed using SPSS statistical software version 21.0 (SPSS Inc.). Quantitative data are presented as mean ± standard deviation. Comparisons between cancer tissues and adjacent non‐cancerous tissues were conducted using paired‐sample *t*‐tests, whereas comparisons between other groups were performed using unpaired *t*‐tests. Multiple group comparisons were made using one‐way analysis of variance (ANOVA) followed by Tukey's post hoc test. Comparisons among different time points for each group were conducted using two‐way ANOVA or repeated measures ANOVA, with Bonferroni adjustments for multiple comparisons. The relationship between miR‐217 and DNMT1 was analyzed using Pearson correlation analysis. A *p*‐value of less than 0.05 was considered statistically significant.

## 3 RESULTS

In this study, we investigated the role and regulatory mechanisms of miR‐217 in the development and progression of DCIS. Bioinformatics analysis suggested that miR‐217 may influence DCIS by modulating the DNMT1/TSHZ2/Hedgehog‐GLI axis. Subsequent in vitro and in vivo experiments demonstrated that miR‐217 suppresses the malignancy and tumorigenesis of DCIS cells by regulating the DNMT1/TSHZ2/Hedgehog‐GLI signaling pathway, highlighting the potential of targeting this axis for novel therapeutic strategies against this disease.

### 3.1 Bioinformatics analysis indicated that miR‐217 might be involved in the development and progression of DCIS by regulating the DNMT1/TSHZ2/Hedgehog‐GLI axis

**FIGURE 1:**
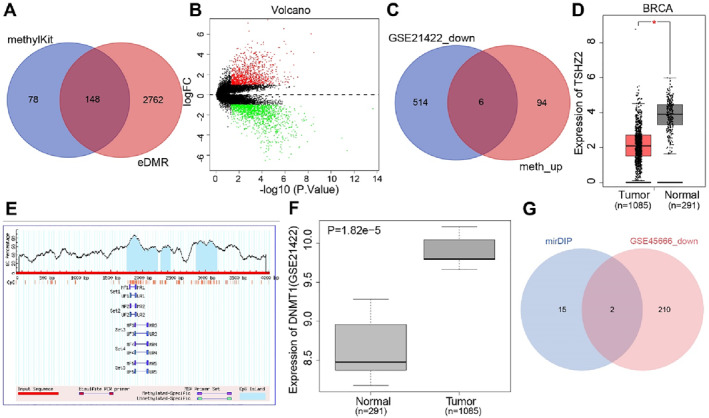
Screening of DCIS‐related genes and prediction of upstream regulatory factors. (A) Venn diagram of differentially methylated genes in promoter regions predicted by the R packages methylKit (left) and eDMR (right); overlapping region indicates shared genes; (B) volcano plot of the GSE21422 microarray for DCIS‐related expression (black: nonsignificant; green: significantly downregulated; red: significantly upregulated); (C) prediction of hypermethylated and low‐expressed genes in DCIS promoter regions (left: top 520 downregulated genes in GSE21422; right: top 100 hypermethylated genes predicted by both methylKit and eDMR); (D) Expression of TSHZ2 in BC samples recorded in the GEPIA database (red: tumor; gray: normal); (E) prediction of CpG islands in the TSHZ2 promoter region by MethPrimer (blue regions represent CpG islands); (F) expression of the DNMT1 gene in the GSE21422 microarray for DCIS‐related expression; (G) prediction of upstream regulatory miRNAs of DNMT1 (blue: miRNAs predicted by mirDIP; red: significantly downregulated miRNAs in BC from GSE45666; overlapping area: common candidates). BC, breast cancer; DCIS, ductal carcinoma in situ; DNMT1, DNA methyltransferase 1; TSHZ2, teashirt zinc finger homeobox 2.

Methylation sequencing data for 24 DCIS tissues and 5 normal tissues were retrieved from the SRA database using RRBS. After quality control, differentially methylated regions were identified using the R packages “methylKit” and “edmr” and annotated with gene and CpG island information (hg19). In DCIS, “methylKit” identified 226 differentially methylated genes in promoter regions (difference = 25, qvalue = 0.01), whereas “edmr” identified 2,910 differentially methylated genes in promoter regions (DMC.qvalue = 0.05, num.DMCs = 1, num.CpGs = 1, DMR.methdiff = 5). The intersection of the genes identified by “methylKit” and “edmr” yielded 148 common genes (Figure 1A). Common genes were extracted from the annotated differentially methylated regions predicted by “methylKit.” The GEO database was then screened for DCIS‐related mRNA expression microarrays (GSE21422). Differential expression analysis, with thresholds of |log_2_FC| > 2 and adjusted *p*‐value < 0.05, identified 1,103 upregulated and 1,377 downregulated mRNAs in DCIS (Figure 1B).

The top 100 hypermethylated genes in promoter regions of DCIS samples were intersected with the top 520 significantly downregulated genes identified from the GSE21422 microarray dataset (Figure 1C). Six overlapping genes were identified in both datasets: GYPC, TSHZ2, KLHL29, TSLP, SEMA3G, and PLAC9. The GEPIA database revealed a significant downregulation of TSHZ2 in BC (Figure 1D).

Predictions generated using the MethPrimer tool identified multiple CpG islands within 2000 bp upstream and downstream of the TSHZ2 promoter region, all exhibiting high methylation levels (Figure 1E). Given the observed hypermethylation of the TSHZ2 promoter and the established role of DNMT1 in maintaining promoter methylation,^17^, ^23^, ^24^ the expression of DNMT1 was analyzed in the DCIS microarray dataset GSE21422. DNMT1 was significantly upregulated in DCIS samples (Figure 1F).

To identify upstream miRNAs that regulate the DNA methyltransferase DNMT1, candidate regulatory miRNAs were predicted using the mirDIP database, which yielded 17 potential targets. Differentially expressed miRNAs associated with BC were then screened from the GEO microarray dataset GSE45666 using the thresholds of |logFC| > 2 and *p*‐value < 0.05. A total of 212 significantly downregulated miRNAs in BC samples were identified. Intersecting these downregulated miRNAs (Table S2) with the 17 candidates predicted by mirDIP (Table S3) revealed two overlapping miRNAs: hsa‐miR‐548k and hsa‐miR‐217 (Figure 1G).

Previous studies have shown that miR‐217 was downregulated in BC and can inhibit tumor progression,^8^ consistent with the present bioinformatics findings. These findings suggest that miR‐217 may influence the progression of BC by regulating DNMT1. Therefore, miR‐217 was selected as a candidate for further experimental investigation.

Previous research has demonstrated that TSHZ2 inhibits the Hedgehog‐GLI signaling pathway,^13^ which has recently been identified as an upregulated molecular target in BC. Activation of this pathway has been observed in human BC cells and is associated with enhanced cell vitality, proliferation, migration, and tumor angiogenesis.^23^, ^24^

In summary, we hypothesize that miR‐217 may mediate the progression of DCIS by promoting the methylation of the TSHZ2 gene promoter region, thereby influencing the Hedgehog‐GLI signaling pathway.

### 3.2 Targeted inhibition of DNMT1 by miR‐217

**FIGURE 2:**
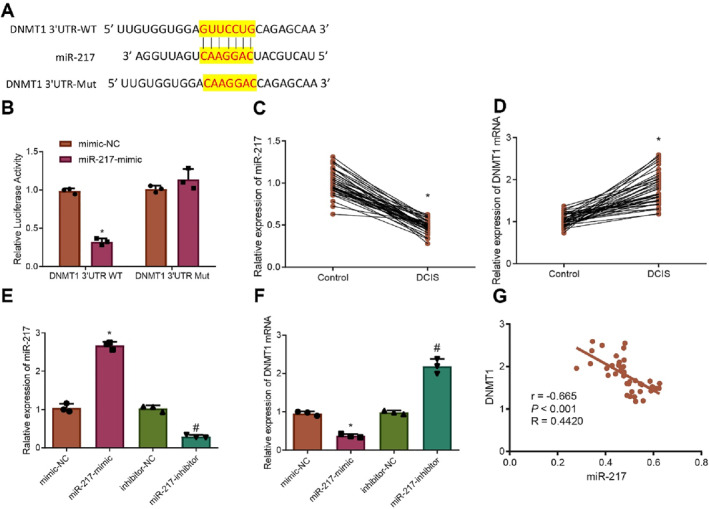
Validation of miR‐217 targeting DNMT1 in ZR‐75‐1 cells. (A) Target binding and mutation sites of miR‐217 and DNMT1. (B) Dual‐luciferase reporter assay validating the targeting relationship between miR‐217 and DNMT1; (C, D) RT‐qPCR analysis of miR‐217 and DNMT1 expression in clinical samples. (E, F) RT‐qPCR analysis of miR‐217 and DNMT1 expression in different cell groups. (G) Correlation analysis of miR‐217 and DNMT1 expression in DCIS samples. Data are presented as mean ± standard deviation, and cell experiments were repeated three times. DCIS, ductal carcinoma in situ; DNMT1, DNA methyltransferase 1; NC, negative control. * indicates *p* < 0.05 compared to the mimic‐NC or Controlgroup; # indicates *p* < 0.05 compared to the inhibitor‐NC group.

Binding and mutation sites of miR‐217 to DNMT1 are illustrated in Figure 2A. Dual‐luciferase reporter assays demonstrated that luciferase activity in the miR‐217‐mimic group co‐transfected with DNMT1 3′UTR WT was significantly reduced compared to the mimic‐NC group. No significant change in luciferase activity was observed in cells transfected with the DNMT1 3′UTR Mut (Figure 2B).

RT‐qPCR analysis of cancerous and adjacent normal tissues from 40 patients with DCIS showed that miR‐217 was significantly downregulated and DNMT1 was significantly upregulated in DCIS tissues compared to normal tissues (Figure 2C,D).

Further intervention of miR‐217 expression in ZR‐75‐1 cells revealed that, relative to the mimic‐NC group, the miR‐217‐mimic group exhibited a significant increase in miR‐217 expression and a decrease in DNMT1 expression. Conversely, compared to the inhibitor‐NC group, the miR‐217‐inhibitor group showed a significant decrease in miR‐217 expression and increased DNMT1 expression (Figure 2E,F). Subsequent correlation analysis of miR‐217 and DNMT1 expressions in DCIS samples revealed a significant negative correlation (Figure 2G).

These findings indicate that miR‐217 is significantly downregulated and DNMT1 is upregulated in DCIS, and miR‐217 can target and inhibit DNMT1 expression.

### 3.3 Inhibition of DCIS cell proliferation, migration, and invasion by miR‐217 via targeted suppression of DNMT1 expression

**FIGURE 3:**
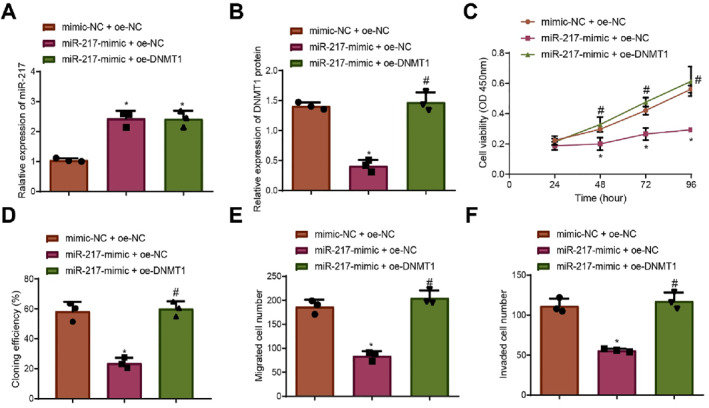
Effect of miR‐217 targeting DNMT1 on DCIS cell proliferation, migration, and invasion. (A) RT‐qPCR analysis of miR‐217 expression in different groups of ZR‐75‐1 cells. (B) Western blot analysis of DNMT1 protein expression in different groups of ZR‐75‐1 cells. (C) CCK‐8 assay for cell proliferation. (D) Monoclonal formation assay for cell clonogenic capacity. (E, F) Transwell assays for cell migration and invasion. Data are presented as mean ± standard deviation, and cell experiments were repeated three times. DCIS, ductal carcinoma in situ; DNMT1, DNA methyltransferase 1; NC, negative control. * indicates *p* < 0.05 compared to themimic‐NC + oe‐NC group; # indicates *p* < 0.05 compared to the miR‐217‐mimic + oe‐NC group.

Expression levels of miR‐217 and DNMT1 were modulated in ZR‐75‐1 cells. RT‐qPCR and Western blot analyses showed that miR‐217 expression significantly increased and DNMT1 expression significantly decreased in the miR‐217‐mimic + oe‐NC group compared to the mimic‐NC + oe‐NC group. Conversely, DNMT1 expression was significantly increased in the miR‐217 + oe‐DNMT1 group, with no significant change in miR‐217 expression compared to the miR‐217‐mimic + oe‐NC group (Figure 3A,B, Figure S1A).

CCK‐8 assays revealed a significant reduction in cell proliferation in the miR‐217‐mimic + oe‐NC group compared to the mimic‐NC + oe‐NC group. In contrast, a significant increase in proliferation was observed in the miR‐217 + oe‐DNMT1 group compared to the miR‐217‐mimic + oe‐NC group (Figure 3C). Monoclonal formation assays showed a significant reduction in clonogenic capacity in the miR‐217‐mimic + oe‐NC group compared to the mimic‐NC + oe‐NC group and a significant increase in the miR‐217 + oe‐DNMT1 group compared to the miR‐217‐mimic + oe‐NC group (Figure 3D, Figure S1B).

Transwell assays indicated that both cell migration and invasion were significantly reduced in the miR‐217‐mimic + oe‐NC group compared to the mimic‐NC + oe‐NC group. Conversely, cell migration and invasion were significantly increased in the miR‐217 + oe‐DNMT1 group compared to the miR‐217‐mimic + oe‐NC group (Figure 3E,F, Figure S1C,D).

Collectively, these findings demonstrate that miR‐217 inhibits DCIS cell proliferation, migration, and invasion by targeting and suppressing DNMT1 expression.

### 3.4 DNMT1 promotes DCIS cell proliferation, migration, and invasion by hypermethylating the TSHZ2 promoter region and suppressing TSHZ2 expression

**FIGURE 4:**
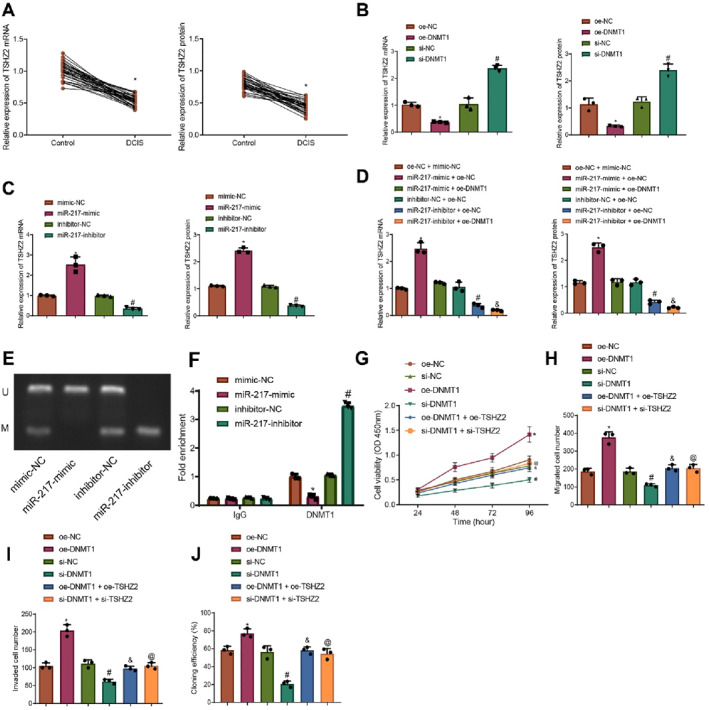
Effect of DNMT1 regulation on TSHZ2 expression and DCIS cell proliferation, migration, and invasion. (A) RT‐qPCR and Western blot analysis of TSHZ2 expression in clinical samples (N: adjacent normal tissues; T: tumor tissues). (B) RT‐qPCR and Western blot analysis of TSHZ2 expression in ZR‐75‐1 cells after DNMT1 expression modulation. (C) RT‐qPCR and Western blot analysis of TSHZ2 expression in ZR‐75‐1 cells after miR‐217 expression modulation; (D) RT‐qPCR and Western blot analysis of TSHZ2 expression in ZR‐75‐1 cells after combined modulation of miR‐217 and DNMT1 expression. (E) Prediction of CpG islands using MethPrimer and MSP analysis of TSHZ2 promoter region methylation. (F) ChIP assay assessing DNMT1 enrichment at the TSHZ2 promoter region. (G) CCK‐8 assay for cell proliferation; (H) Monoclonal formation assay for clonogenic capacity. (I, J) Transwell assay for cell migration and invasion. Data are presented as mean ± standard deviation, and cell experiments were repeated three times. ChIP, chromatin immunoprecipitation; DCIS, ductal carcinoma in situ; DNMT1, DNA methyltransferase 1; MSP, methylation‐specific PCR; NC, negative control; TSHZ2, teashirt zinc finger homeobox 2. * indicates *p* < 0.05 comparedto the Control group, oe‐NC group, mimic‐NC group, or oe‐NC + mimic‐NC group; # indicates *p* < 0.05 compared to the si‐NCgroup, inhibitor‐NC group, or inhibitor‐NC + oe‐NC group; & indicates *p* < 0.05 compared to the miR‐217‐inhibitor + oe‐NC group or oe‐DNMT1 group; @ indicates *p* < 0.05 compared to the si‐DNMT1group.

Bioinformatics analysis suggested that DNMT1 may contribute to the development and progression of DCIS by promoting methylation of the TSHZ2 gene promoter region. Subsequently, TSHZ2 expression was measured in tumor and adjacent normal tissues from 40 DCIS patients using RT‐qPCR and Western blot. The results indicated that TSHZ2 expression was significantly lower in DCIS tissues compared to normal tissues (Figure 4A, Figure S2A). Next, DNMT1 expression was modulated in ZR‐75‐1 cells, and TSHZ2 expression was assessed. The results showed that overexpression of DNMT1 significantly reduced TSHZ2 expression in ZR‐75‐1 cells, whereas knockdown of DNMT1 significantly increased TSHZ2 expression (Figure 4B, Figure S2B).

In ZR‐75‐1 cells, expression of miR‐217 was modulated, and TSHZ2 expression was analyzed by RT‐qPCR and Western blot. Compared to the mimic‐NC group, TSHZ2 expression was significantly upregulated in the miR‐217‐mimic group. In contrast, TSHZ2 expression was significantly decreased in the miR‐217‐inhibitor group relative to the inhibitor‐NC group (Figure 4C, Figure S2C). Further, co‐modulation of miR‐217 and DNMT1 in ZR‐75‐1 cells showed that TSHZ2 expression was significantly reduced in the miR‐217‐mimic + oe‐DNMT1 group compared to the miR‐217 + oe‐NC group. Similarly, TSHZ2 expression was significantly lower in the miR‐217‐inhibitor + oe‐DNMT1 group compared to the miR‐217‐inhibitor + oe‐NC group (Figure 4D, Figure S2D).

MSP assays were conducted to evaluate the methylation status of the TSHZ2 gene promoter region. Partial methylation at specific sites was detected in the mimic‐NC and inhibitor‐NC groups. In contrast, no methylation was observed in the miR‐217‐mimic group, whereas enhanced methylation was observed in the miR‐217‐inhibitor group (Figure 4E). ChIP assays were performed to assess the enrichment of DNMT1 in the TSHZ2 promoter region. Compared to the mimic‐NC group, DNMT1 enrichment was significantly decreased in the miR‐217‐mimic group, whereas DNMT1 enrichment was significantly increased in the miR‐217‐inhibitor group compared to the inhibitor‐NC group (Figure 4F). These findings demonstrate that miR‐217 can modulate the expression of TSHZ2 by targeting DNMT1, thereby reducing its enrichment at the TSHZ2 promoter region and consequently upregulating TSHZ2 expression.

The impact of DNMT1‐mediated regulation of TSHZ2 expression on the biological behavior of DCIS cells was further investigated. CCK‐8 assays, monoclonal formation tests, and Transwell assays demonstrated that cells in the oe‐DNMT1 group exhibited significantly enhanced proliferation, clonogenic capacity, migration, and invasion. Conversely, the si‐DNMT1 group displayed significant reductions in these activities compared to the si‐NC group. Additionally, the oe‐DNMT1 + oe‐TSHZ2 group exhibited significant decreases in these cellular functions compared to the oe‐DNMT1 group alone. Similarly, the si‐DNMT1 + si‐TSHZ2 group showed significant increases compared to the si‐DNMT1 group (Figure 4G,J, Figure S2E,G).

These findings indicate that DNMT1 promotes DCIS cell proliferation, migration, and invasion by inducing hypermethylation of the TSHZ2 promoter region, thereby suppressing TSHZ2 expression.

### 3.5 Inhibition of DCIS cell proliferation, migration, and invasion by TSHZ2 through the suppression of the Hedgehog‐GLI signaling pathway

**FIGURE 5:**
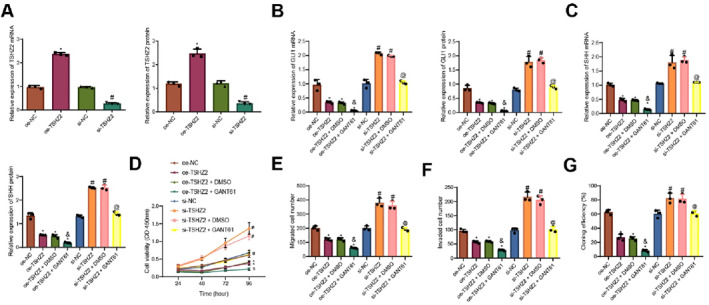
Effect of TSHZ2 regulation on the Hedgehog‐GLI signaling pathway and DCIS cell proliferation, migration, and invasion. (A) RT‐qPCR and Western blot analysis of TSHZ2 transfection efficiency in ZR‐75‐1 cells after TSHZ2 expression modulation. (B, C) RT‐qPCR and Western blot analysis of Hedgehog‐GLI pathway‐related factors (GLI1 and SHH) in different cell groups. (D) CCK‐8 assay for evaluating cell proliferation. (E) Monoclonal formation assay for assessing clonogenic capacity; (F, G) Transwell assay for measuring cell migration and invasion. Data are presented as mean ± standard deviation, and cell experiments were repeated three times. DCIS, ductal carcinoma in situ; NC, negative control; SHH, Sonic Hedgehog; TSHZ2, teashirt zinc finger homeobox 2. * indicates *p* < 0.05 compared to the oe‐NC group; # indicates *p* < 0.05 compared to the si‐NC group; & indicates *p* < 0.05 compared to the oe‐TSHZ2 + DMSO group; @ indicates *p* < 0.05compared to the si‐TSHZ2 + DMSO group.

We further investigated the impact of TSHZ2 regulation on the Hedgehog‐GLI signaling pathway in DCIS cell biology. Expression of TSHZ2 was modulated in ZR‐75‐1 cells, and transfection efficiency was confirmed by RT‐qPCR and Western blot (Figure 5A). Cells with altered TSHZ2 expression were subsequently treated with the Hedgehog‐GLI pathway inhibitor GANT61. RT‐qPCR and Western blot were used to detect the expression of Hedgehog‐GLI pathway‐related factors (GLI1 and SHH) in each group. Compared to the oe‐NC group, GLI1 and SHH expression were significantly reduced in the oe‐TSHZ2 group. Furthermore, GLI1 and SHH expression were significantly lower in the oe‐TSHZ2 + GANT61 group compared to the oe‐TSHZ2 + DMSO group. Conversely, GLI1 and SHH expression were significantly increased in the si‐TSHZ2 group compared to the si‐NC group but significantly decreased in the si‐TSHZ2 + GANT61 group compared to the si‐TSHZ2 + DMSO group (Figure 5B,C).

CCK‐8, monoclonal formation and Transwell assay results demonstrated that the oe‐TSHZ2 group exhibited significantly reduced cell proliferation, clonogenic capacity, migration, and invasion compared to the oe‐NC group. Furthermore, the oe‐TSHZ2 + GANT61 group showed further reductions in these cellular activities compared to the oe‐TSHZ2 + DMSO group. Conversely, the si‐TSHZ2 group exhibited significant increases in cell proliferation, clonogenic capacity, migration, and invasion compared to the si‐NC group. These activities were significantly reduced in the si‐TSHZ2 + GANT61 group compared to the si‐TSHZ2 + DMSO group (Figure 5D–G).

These results indicate that TSHZ2 inhibits DCIS cell proliferation, migration, and invasion by suppressing the Hedgehog‐GLI signaling pathway.

### 3.6 miR‐217 inhibits tumor formation in vivo by targeting DNMT1 to promote TSHZ2 expression and suppress the Hedgehog‐GLI signaling pathway

**FIGURE 6:**
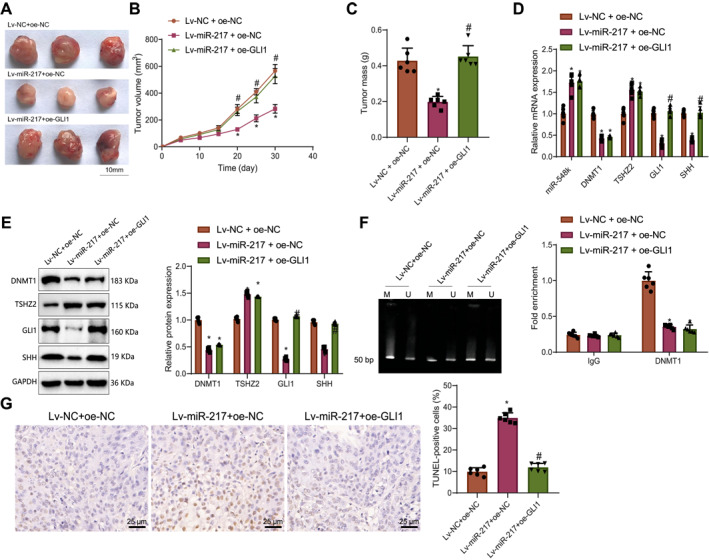
Effect of miR‐217 on DNMT1/TSHZ2/Hedgehog‐GLI axis and tumor formation in DCIS cells in vivo. (A) Representative images of tumors from each group of nude mice. (B) Comparison of tumor volumes among different groups of nude mice. (C) Comparison of tumor masses among different groups of nude mice; (D, E) RT‐qPCR and Western blot analysis of miR‐217, DNMT1, TSHZ2, GLI1, and SHH expression in tumor tissues from different groups of nude mice. (F) MSP analysis of TSHZ2 promoter region methylation and ChIP analysis of DNMT1 enrichment at the TSHZ2 promoter region. (G) TUNEL staining for measuring apoptosis in tumor tissues from different groups of nude mice. Data are presented as mean ± standard deviation. *n* = 6. ChIP, chromatin immunoprecipitation; DCIS, ductal carcinoma in situ; DNMT1, DNA methyltransferase 1; MSP, methylation‐specific PCR; NC, negative control; SHH, Sonic Hedgehog; TSHZ2, teashirt zinc finger homeobox 2. * indicates *p* < 0.05 compared to theLv‐NC + oe‐NC group; # indicates *p* < 0.05 compared to the Lv‐miR‐217 + oe‐NC group.

Next, we investigated the effect miR‐217 on the DNMT1/TSHZ2/Hedgehog‐GLI axis in vivo using a DCIS cell nude mouse xenograft model. Tumor volume and mass were monitored and measured, revealing significant reductions in the Lv‐miR‐217 + oe‐NC group compared to the Lv‐NC + oe‐NC group. Conversely, tumor volume and mass significantly increased in the Lv‐miR‐217 + oe‐GLI1 group compared to the Lv‐miR‐217 + oe‐NC group (Figure 6A–C).

RT‐qPCR and Western blot analyses showed that miR‐217 and TSHZ2 expressions were significantly increased in the tumor tissues of the Lv‐miR‐217 + oe‐NC group compared to the Lv‐NC + oe‐NC group, whereas DNMT1, SHH, and GLI1 expression were significantly decreased. Additionally, SHH and GLI1 expression were significantly higher in the Lv‐miR‐217 + oe‐GLI1 group compared to the Lv‐miR‐217 + oe‐NC group (Figure 6D,E).

MSP and ChIP assays were conducted to assess the impact of DNMT1 on the methylation of the TSHZ2 promoter in tumor tissues. The results showed that DNMT1 enrichment at the TSHZ2 promoter region was significantly reduced in the Lv‐miR‐217 + oe‐NC group compared to the Lv‐NC + oe‐NC group (Figure 6F).

TUNEL staining was used to detect apoptosis in tumor tissues from nude mice. The results indicated that apoptosis was significantly increased in the Lv‐miR‐217 + oe‐NC group compared to the Lv‐NC + oe‐NC group. Conversely, apoptosis was significantly decreased in the Lv‐miR‐217 + oe‐GLI1 group compared to the Lv‐miR‐217 + oe‐NC group (Figure 6G).

These findings suggest that miR‐217 inhibits in vivo tumor formation by targeting and suppressing DNMT1, promoting TSHZ2 expression and inhibiting the Hedgehog‐GLI signaling pathway.

## 4 DISCUSSION

DCIS is recognized as a precursor to invasive BC, accounting for 25% of all BC diagnoses and with the potential to progress to invasive BC.^25^ The present findings demonstrate that miR‐217 inhibits the malignant characteristics of DCIS cells by regulating the DNMT1/TSHZ2/Hedgehog‐GLI signaling axis, highlighting its potential as a novel targeted therapy for DCIS.

Previous studies have demonstrated that miRNAs interact with specific target mRNAs at the 3′ UTR, inhibiting their expression.^26^ This study identified miR‐217 as a direct regulator of DNMT1, likely reducing its expression. These findings provide evidence for the post‐transcriptional regulation of DNMT1 by miR‐217 in DCIS and underscore its importance in modulating DCIS progression. Furthermore, our research revealed that miR‐217 can inhibit the malignant properties of DCIS cells by suppressing DNMT1. miR‐548‐3p is significantly downregulated in BC, and its overexpression can inhibit the malignant potential of BC cells. Similarly, inhibition of DNMT1 activity has been reported to reduce BC cell proliferation in vitro and tumor growth in vivo. These findings suggest that miR‐217‐mediated downregulation of DNMT1 could serve as a potential therapeutic target for DCIS.

Our study further demonstrated that DNMT1 induces hypermethylation of the TSHZ2 promoter region, thereby inhibiting TSHZ2 expression and promoting cell proliferation and metastasis. Consistently, DNMT1 has been shown to enhance the metastatic and invasive phenotype of TNBC cells by inducing hypermethylation of multiple tumor suppressor gene promoters. Hypermethylation in specific regions is associated with the transcriptional silencing of certain tumor suppressor genes. In addition to the downregulation of TSHZ2 expression, the TSHZ2 promoter has been found to be methylated in the BC cell line MDA‐MB‐231. Moreover, reduced TSHZ2 expression in BC cells promotes breast tumorigenesis through the activation of GLI1, which aligns with our findings that TSHZ2 can inactivate the Hedgehog‐GLI signaling pathway, thereby reducing DCIS cell proliferation and metastasis. Previous studies have also established that the Hedgehog‐GLI signaling pathway is primarily activated in human BC cells, leading to increased cancer cell viability, proliferation, and migration.^23^

Additionally, activation of the Hedgehog‐GLI pathway has been implicated in the initiation and progression of BC tumors. Therefore, TSHZ2‐induced inactivation of this pathway may serve as a potential therapeutic target for DCIS. It is worth noting that TSHZ2 has been identified as a key biomarker for predicting BC survival, and its regulatory mechanisms are not limited to the miR‐217/DNMT1 pathway identified in this study. For instance, recent research has demonstrated that the EGF/DDGs/TSHZ2 axis can influence BC metastasis. Further investigation into the interactions and regulatory mechanisms among these molecular targets will be prioritized in future research.

Although the present study is the first to confirm that miR‐217 regulates DNMT1/TSHZ2/Hedgehog‐GLI signaling to modulate BC tumorigenesis, several limitations remain. First, TSHZ2 is a critical biomarker of BC survival, and its regulatory mechanisms are not limited to the miR‐217/DNMT1 axis described herein. For example, recent research has shown that EGF/DDGs/TSHZ2 axis influences BC metastasis. Interactions and reciprocal regulation among these molecular pathways will be explored in future investigations. Additionally, in vivo results suggest that miR‐217 suppresses TSHZ2 promoter methylation by targeting DNMT1, thereby inhibiting Hedgehog‐GLI signaling, suppressing tumorigenesis, and promoting apoptosis. Although emerging evidence supports that miRNAs can suppress Hedgehog‐GLI activation, the specific role of miR‐217 in this pathway requires further study due to limited supporting literature. Finally, further clinical research is needed to validate miR‐217's mechanism of action in BC. Such studies will be essential for translating these findings from cellular and animal models into future clinical applications.

## 5 CONCLUSION

In summary, this study demonstrates that miR‐217 acts as a critical regulatory factor in the biology of DCIS cells by targeting DNMT1, inhibiting methylation of the TSHZ2 promoter region, and suppressing the Hedgehog‐GLI signaling pathway. Therefore, the miR‐217/DNMT1/TSHZ2/Hedgehog‐GLI axis represents a potential therapeutic target for DCIS and related diseases.

## AUTHOR CONTRIBUTIONS

Z.W. and L.W. conceived and designed the study. S.L. and S.G. performed the experiments. Z.W. and L.W. analyzed the data. Z.W. and X.L. wrote the manuscript. All authors reviewed and approved the final version of the manuscript.

## CONFLICT OF INTEREST STATEMENT

The author declares no conflicts of interest.

## ETHICS STATEMENT

This study was approved by the Clinical Ethics Committee of Qiqihar Medical University (Approval No. 2022‐65). All animal experiments were approved by the Animal Ethics Committee of Qiqihar Medical University (Approval No. QMU‐AECC‐2022‐83).

## Supporting information

Supporting Information S1

## Data Availability

All data can be provided as needed.
