# Supplementary material for: Epigenetic regulation of breast ductal carcinoma in situ by miR‐217 through DNMT1 and Hedgehog‐GLI pathway
Source: J Cell Commun Signal. 2025 Sep 3;19(3):e70030. doi: 10.1002/ccs3.70030 (PMC12408178; doi:10.1002/ccs3.70030)
Supplement: Supplementary file 1 — Supporting Information S1 [file CCS3-19-e70030-s001.docx]

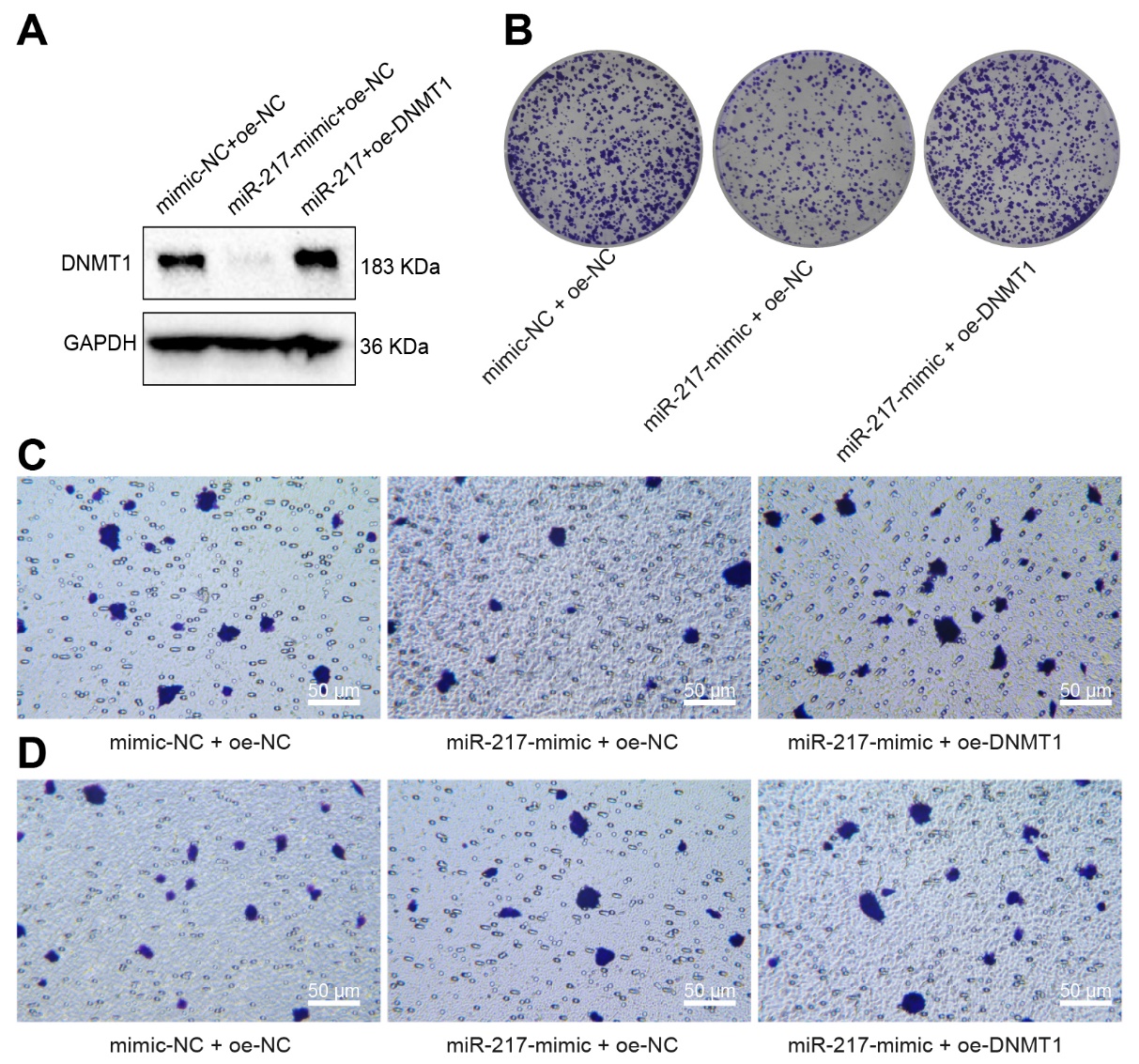


**Figure S1. Effect of miR-217 targeting DNMT1 on DCIS cell proliferation, migration, and invasion.**

Note: (A) Western blot analysis of DNMT1 protein levels in different groups; (B) Monoclonal formation assay evaluating the clonogenic capacity of ZR-75-1 cells under various treatments; (C-D) Transwell assay assessing the impact of different treatments on ZR-75-1 cell migration and invasion.


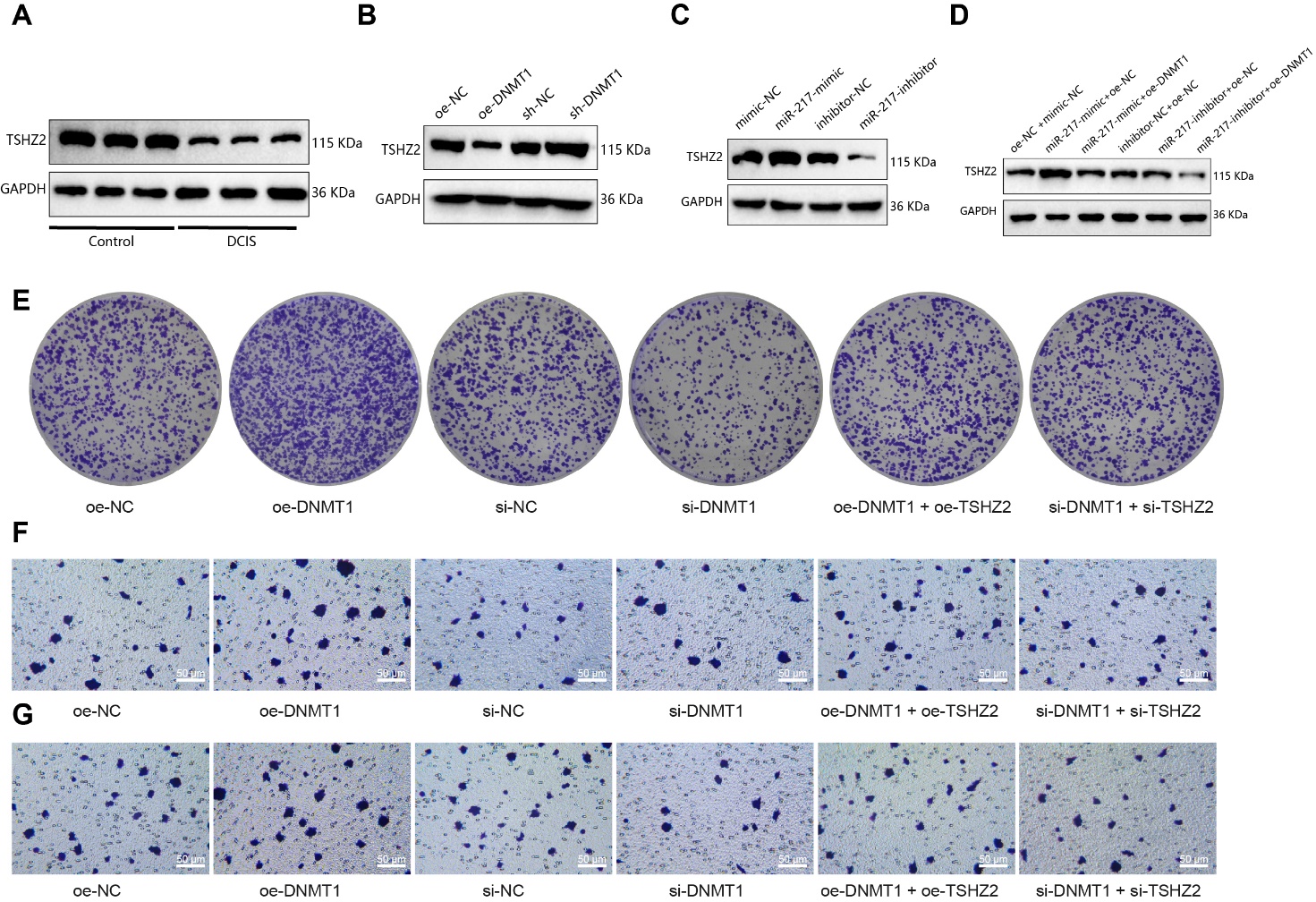


**Figure S2. Effect of DNMT1 regulation on TSHZ2 expression and DCIS cell proliferation, migration, and invasion.**

Note: (A) Western blot analysis of TSHZ2 expression in different tissue groups; (B-D) Western blot analysis of TSHZ2 expression in different cell groups; (E) Monoclonal formation assay evaluating the clonogenic capacity of ZR-75-1 cells under various treatments; (F-G) Transwell assay assessing the impact of different treatments on ZR-75-1 cell migration and invasion.

**Table S1. RT-qPCR primer sequence.**

| **Gene** | **Primer sequence (5'-3')** |
| --- | --- |
| miR-217 | F: 5'-TACTGCATCAGGAACTGA-3' |
|  | R: Universal reverse sequence of reagent kit |
| DNMT1 | F: 5'-CCATCAGGCATTCTACCA-3' |
|  | R: 5'-CGTTCTCCTTGTCTTCTCT-3' |
| TSHZ2 | F: 5'-GGACGAGGAGCTAGAAACGG-3' |
|  | R: 5'-CTCTTGATGTCCGACACCTGA-3' |
| GLI1 | F: 5'-TACATCAACTCCGGCCAATAGG-3'  R: 5'-CGGCGGCTGACAGTATAGGCA-3' |
| SHH | F: 5-GTGGCCGAGAAGACCCTA-3  R: 5'-CAAAGCGTTCAACTTGTCCTTA-3' |
| GAPDH | F: 5-ACCACAGTCCATGCCATCAC-3 |
|  | R: 5-TCCACCACCCTGTTGCTGTA-3 |
| U6 | F: 5-GGGCAGGAAGAGGGCCTAT-3 |
|  | R: 5-GGCAGGAAGAGGGCCTAT-3 |

**Table S2. The significantly downregulated miRNAs in breast cancer samples from the GSE45666 microarray dataset**

| **miRNA_ID** | **logFC** | **P.Value** | **adj.P.Val** |
| --- | --- | --- | --- |
| hsa-miR-139-5p | -4.058138 | 1.09e-21 | 3.90e-18 |
| hsa-miR-99a | -3.084548 | 1.98e-17 | 2.34e-14 |
| hsa-miR-99a | -3.056333 | 3.19e-17 | 2.34e-14 |
| hsa-miR-99a | -3.056081 | 3.35e-17 | 2.34e-14 |
| hsa-miR-99a | -3.043677 | 3.36e-17 | 2.34e-14 |
| hsa-miR-548k | -2.988951 | 4.40e-17 | 2.34e-14 |
| hsa-miR-99a | -3.007254 | 4.58e-17 | 2.34e-14 |
| hsa-miR-99a | -3.005303 | 5.81e-17 | 2.40e-14 |
| hsa-miR-99a | -2.97788 | 6.05e-17 | 2.40e-14 |
| hsa-miR-195 | -2.577472 | 7.93e-17 | 2.83e-14 |
| hsa-miR-195 | -2.601504 | 1.04e-16 | 3.36e-14 |
| hsa-miR-483-3p | -2.418442 | 1.75e-16 | 5.21e-14 |
| hsa-miR-195 | -2.426678 | 2.10e-16 | 5.51e-14 |
| hsa-miR-195 | -2.425895 | 2.32e-16 | 5.51e-14 |
| hsa-miR-195 | -2.42027 | 2.58e-16 | 5.51e-14 |
| hsa-miR-195 | -2.420454 | 2.60e-16 | 5.51e-14 |
| hsa-miR-125b-2* | -2.575391 | 2.65e-16 | 5.51e-14 |
| hsa-miR-195 | -2.416303 | 2.78e-16 | 5.51e-14 |
| hsa-miR-195 | -2.567646 | 3.68e-16 | 6.62e-14 |
| hsa-miR-217 | -2.408342 | 3.82e-16 | 6.62e-14 |
| hsa-miR-195 | -2.564399 | 3.90e-16 | 6.62e-14 |
| hsa-miR-195 | -2.532354 | 5.10e-16 | 8.26e-14 |
| hsa-miR-195 | -2.508407 | 7.50e-16 | 1.16e-13 |
| hsa-miR-125b-2* | -2.459703 | 8.01e-16 | 1.19e-13 |
| hsa-miR-195 | -2.407673 | 1.11e-15 | 1.58e-13 |
| hsa-miR-195 | -2.509245 | 1.15e-15 | 1.58e-13 |
| hsa-miR-497 | -2.31562 | 1.27e-15 | 1.68e-13 |
| hsa-miR-195 | -2.481 | 1.49e-15 | 1.90e-13 |
| hsa-miR-125b | -2.911445 | 1.58e-15 | 1.94e-13 |
| hsa-miR-195 | -2.407483 | 1.69e-15 | 2.01e-13 |
| hsa-miR-497 | -2.223175 | 2.49e-15 | 2.86e-13 |
| hsa-miR-497 | -2.225407 | 2.62e-15 | 2.92e-13 |
| hsa-miR-99a | -2.884327 | 4.01e-15 | 4.33e-13 |
| hsa-miR-497 | -2.163125 | 4.96e-15 | 5.09e-13 |
| hsa-miR-99a | -2.943508 | 5.20e-15 | 5.09e-13 |
| hsa-miR-497 | -2.164156 | 5.24e-15 | 5.09e-13 |
| hsa-miR-125b | -2.80545 | 5.29e-15 | 5.09e-13 |
| hsa-miR-99a | -2.90772 | 7.66e-15 | 7.07e-13 |
| hsa-miR-125b | -2.78074 | 7.73e-15 | 7.07e-13 |
| hsa-miR-125b | -2.784256 | 7.97e-15 | 7.11e-13 |
| hsa-miR-497 | -2.153879 | 8.58e-15 | 7.46e-13 |
| hsa-miR-125b | -2.780679 | 9.67e-15 | 8.21e-13 |
| hsa-miR-99a | -2.846468 | 1.26e-14 | 1.05e-12 |
| hsa-miR-99a | -2.806529 | 1.57e-14 | 1.27e-12 |
| hsa-miR-497 | -2.248255 | 1.70e-14 | 1.34e-12 |
| hsa-miR-125b | -2.760464 | 1.73e-14 | 1.34e-12 |
| hsa-miR-125b | -2.733445 | 1.98e-14 | 1.47e-12 |
| hsa-miR-99a | -2.732926 | 1.98e-14 | 1.47e-12 |
| hsa-miR-125b | -2.786498 | 2.58e-14 | 1.88e-12 |
| hsa-miR-99a | -2.789434 | 2.64e-14 | 1.88e-12 |
| hsa-miR-99a | -2.747942 | 9.21e-14 | 6.44e-12 |
| hsa-miR-10b | -2.30652 | 1.52e-13 | 1.04e-11 |
| hsa-miR-10b | -2.228086 | 2.12e-13 | 1.43e-11 |
| hsa-miR-125b-2* | -2.602029 | 2.39e-13 | 1.58e-11 |
| hsa-miR-497 | -2.133253 | 3.35e-13 | 2.13e-11 |
| hsa-miR-100 | -2.759842 | 3.96e-13 | 2.48e-11 |
| hsa-miR-10b | -2.223253 | 4.37e-13 | 2.69e-11 |
| hsa-miR-10b | -2.289315 | 5.07e-13 | 3.06e-11 |
| hsa-miR-100 | -2.730541 | 5.37e-13 | 3.19e-11 |
| hsa-miR-10b | -2.169198 | 5.89e-13 | 3.42e-11 |
| hsa-miR-497 | -2.20941 | 5.95e-13 | 3.42e-11 |
| hsa-miR-10b | -2.147408 | 6.35e-13 | 3.59e-11 |
| hsa-miR-497 | -2.05954 | 6.72e-13 | 3.74e-11 |
| hsa-miR-100 | -2.598368 | 8.16e-13 | 4.41e-11 |
| hsa-miR-10b | -2.191527 | 1.17e-12 | 6.02e-11 |
| hsa-miR-497 | -2.045647 | 1.37e-12 | 6.97e-11 |
| hsa-miR-10b | -2.189129 | 1.53e-12 | 7.70e-11 |
| hsa-miR-497 | -2.022778 | 1.62e-12 | 8.05e-11 |
| hsa-miR-497 | -2.546414 | 1.99e-12 | 9.73e-11 |
| hsa-miR-10b | -2.191246 | 2.03e-12 | 9.77e-11 |
| hsa-miR-10b* | -2.107106 | 2.29e-12 | 1.05e-10 |
| hsa-miR-10b | -2.125232 | 2.33e-12 | 1.05e-10 |
| hsa-miR-497 | -2.022365 | 2.34e-12 | 1.05e-10 |
| hsa-miR-10b | -2.157452 | 2.36e-12 | 1.05e-10 |
| hsa-miR-497 | -2.040021 | 2.45e-12 | 1.07e-10 |
| hsa-miR-100 | -2.591419 | 2.71e-12 | 1.15e-10 |
| hsa-miR-497 | -2.027352 | 3.28e-12 | 1.36e-10 |
| hsa-miR-100 | -2.537469 | 3.91e-12 | 1.60e-10 |
| hsa-miR-145* | -2.30959 | 4.31e-12 | 1.73e-10 |
| hsa-miR-10b | -2.268763 | 4.94e-12 | 1.87e-10 |
| hsa-miR-10b | -2.074659 | 5.23e-12 | 1.96e-10 |
| hsa-miR-100 | -2.493904 | 5.36e-12 | 1.99e-10 |
| hsa-miR-100 | -2.491004 | 5.43e-12 | 1.99e-10 |
| hsa-miR-10b | -2.065506 | 6.44e-12 | 2.30e-10 |
| hsa-miR-10b | -2.085846 | 6.50e-12 | 2.30e-10 |
| hsa-miR-145* | -2.226905 | 6.67e-12 | 2.33e-10 |
| hsa-miR-125b | -3.434979 | 7.27e-12 | 2.49e-10 |
| hsa-miR-145* | -2.238567 | 9.01e-12 | 3.06e-10 |
| hsa-miR-145* | -2.310953 | 9.88e-12 | 3.29e-10 |
| hsa-miR-10b | -2.03252 | 1.00e-11 | 3.32e-10 |
| hsa-miR-100 | -2.558337 | 1.02e-11 | 3.32e-10 |
| hsa-miR-145* | -2.206389 | 1.50e-11 | 4.56e-10 |
| hsa-miR-145* | -2.287308 | 1.52e-11 | 4.56e-10 |
| hsa-miR-125b | -2.56568 | 2.00e-11 | 5.79e-10 |
| hsa-miR-145* | -2.254579 | 2.15e-11 | 6.19e-10 |
| hsa-miR-125b | -2.636217 | 2.39e-11 | 6.76e-10 |
| hsa-miR-125b | -2.81499 | 2.79e-11 | 7.58e-10 |
| hsa-miR-125b | -2.721006 | 2.98e-11 | 7.94e-10 |
| hsa-miR-125b | -2.872444 | 3.41e-11 | 8.87e-10 |
| hsa-miR-601 | -2.321251 | 3.44e-11 | 8.89e-10 |
| hsa-miR-601 | -2.409352 | 3.94e-11 | 9.96e-10 |
| hsa-miR-601 | -2.110115 | 5.66e-11 | 1.36e-09 |
| hsa-miR-125b | -2.625439 | 6.03e-11 | 1.42e-09 |
| hsa-miR-601 | -2.324321 | 6.72e-11 | 1.53e-09 |
| hsa-miR-126* | -2.372048 | 7.99e-11 | 1.79e-09 |
| hsa-miR-601 | -2.266149 | 8.12e-11 | 1.80e-09 |
| hsa-miR-601 | -2.225685 | 1.10e-10 | 2.35e-09 |
| hsa-miR-601 | -2.368168 | 1.33e-10 | 2.78e-09 |
| hsa-miR-601 | -2.332047 | 1.60e-10 | 3.27e-09 |
| hsa-miR-125b | -2.950614 | 1.95e-10 | 3.81e-09 |
| hsa-miR-601 | -2.23232 | 1.97e-10 | 3.83e-09 |
| hsa-miR-126* | -2.294515 | 2.18e-10 | 4.17e-09 |
| hsa-miR-145 | -2.594471 | 2.76e-10 | 5.07e-09 |
| hsa-miR-145 | -2.541979 | 2.94e-10 | 5.34e-09 |
| hsa-miR-100 | -2.576833 | 2.98e-10 | 5.40e-09 |
| hsa-miR-145 | -2.505259 | 3.11e-10 | 5.58e-09 |
| hsa-miR-601 | -2.140684 | 3.64e-10 | 6.23e-09 |
| hsa-miR-145 | -2.473257 | 4.20e-10 | 7.06e-09 |
| hsa-miR-601 | -2.192136 | 4.30e-10 | 7.17e-09 |
| hsa-miR-145 | -2.940338 | 4.36e-10 | 7.18e-09 |
| hsa-miR-145 | -2.480575 | 4.50e-10 | 7.29e-09 |
| hsa-miR-154 | -2.179205 | 4.59e-10 | 7.37e-09 |
| hsa-miR-486-5p | -2.067161 | 8.05e-10 | 1.21e-08 |
| hsa-miR-145 | -2.637926 | 8.41e-10 | 1.25e-08 |
| hsa-miR-486-5p | -2.056437 | 8.91e-10 | 1.31e-08 |
| hsa-miR-145 | -3.229709 | 1.22e-09 | 1.70e-08 |
| hsa-miR-154 | -2.213371 | 1.30e-09 | 1.79e-08 |
| hsa-miR-601 | -2.225465 | 1.31e-09 | 1.80e-08 |
| hsa-miR-486-5p | -2.138885 | 1.34e-09 | 1.82e-08 |
| hsa-miR-30c | -2.105617 | 1.40e-09 | 1.89e-08 |
| hsa-miR-30a* | -2.488069 | 1.43e-09 | 1.93e-08 |
| hsa-miR-486-5p | -2.047925 | 1.48e-09 | 1.97e-08 |
| hsa-miR-30c | -2.178164 | 1.62e-09 | 2.13e-08 |
| hsa-miR-145 | -3.038059 | 1.64e-09 | 2.14e-08 |
| hsa-miR-299-5p | -2.17947 | 1.87e-09 | 2.42e-08 |
| hsa-miR-486-5p | -2.087164 | 2.39e-09 | 2.99e-08 |
| hsa-miR-100 | -2.41674 | 2.46e-09 | 3.05e-08 |
| hsa-miR-299-5p | -2.28669 | 2.84e-09 | 3.46e-08 |
| hsa-miR-154 | -2.114968 | 2.86e-09 | 3.47e-08 |
| hsa-miR-154 | -2.189811 | 3.05e-09 | 3.66e-08 |
| hsa-miR-486-5p | -2.078997 | 3.38e-09 | 3.96e-08 |
| hsa-miR-30a* | -2.493181 | 3.91e-09 | 4.44e-08 |
| hsa-miR-486-5p | -2.11037 | 3.96e-09 | 4.47e-08 |
| hsa-miR-126* | -2.033027 | 4.19e-09 | 4.70e-08 |
| hsa-miR-145 | -3.085497 | 4.65e-09 | 5.14e-08 |
| hsa-miR-100 | -2.368518 | 5.00e-09 | 5.46e-08 |
| hsa-miR-100 | -2.442267 | 5.18e-09 | 5.64e-08 |
| hsa-miR-601 | -2.347868 | 5.23e-09 | 5.67e-08 |
| hsa-miR-100 | -2.392537 | 5.39e-09 | 5.77e-08 |
| hsa-miR-154 | -2.134527 | 5.77e-09 | 6.12e-08 |
| hcmv-miR-UL70-3p | -2.076958 | 6.24e-09 | 6.47e-08 |
| hsa-miR-100 | -2.297881 | 6.78e-09 | 6.94e-08 |
| hsa-miR-486-5p | -2.045255 | 6.90e-09 | 7.01e-08 |
| hcmv-miR-UL70-3p | -2.082681 | 8.39e-09 | 8.36e-08 |
| hsa-miR-30a* | -2.386175 | 8.87e-09 | 8.71e-08 |
| hsa-miR-30a* | -2.373413 | 1.02e-08 | 9.86e-08 |
| hsa-miR-30a* | -2.333116 | 1.67e-08 | 1.48e-07 |
| hsa-miR-100 | -2.425109 | 1.86e-08 | 1.63e-07 |
| hsa-miR-1202 | -2.49608 | 1.93e-08 | 1.68e-07 |
| hsa-miR-100 | -2.435984 | 1.94e-08 | 1.69e-07 |
| hsa-miR-299-5p | -2.214253 | 2.06e-08 | 1.77e-07 |
| hsa-miR-30a* | -2.383761 | 2.27e-08 | 1.93e-07 |
| hsa-miR-30a* | -2.298194 | 2.28e-08 | 1.94e-07 |
| hsa-miR-30a* | -2.327706 | 2.39e-08 | 2.02e-07 |
| hsa-miR-1202 | -2.398809 | 2.79e-08 | 2.30e-07 |
| hsa-miR-1202 | -2.444043 | 2.80e-08 | 2.30e-07 |
| hsa-miR-30a* | -2.279319 | 2.92e-08 | 2.38e-07 |
| hsa-miR-30a* | -2.475636 | 3.08e-08 | 2.48e-07 |
| hsa-miR-1202 | -2.309023 | 3.60e-08 | 2.84e-07 |
| hsa-miR-30a* | -2.263748 | 5.03e-08 | 3.82e-07 |
| hsa-miR-30a* | -2.349006 | 5.09e-08 | 3.86e-07 |
| hsa-miR-30a* | -2.283453 | 5.18e-08 | 3.90e-07 |
| hsa-miR-1202 | -2.262293 | 6.18e-08 | 4.53e-07 |
| hsa-miR-1202 | -2.248297 | 6.65e-08 | 4.82e-07 |
| hsa-miR-30a* | -2.251586 | 6.89e-08 | 4.96e-07 |
| hsa-miR-199b-5p | -2.554723 | 7.87e-08 | 5.55e-07 |
| hsa-miR-30a* | -2.318256 | 8.05e-08 | 5.64e-07 |
| hsa-miR-1202 | -2.260074 | 8.13e-08 | 5.68e-07 |
| hsa-miR-1202 | -2.224702 | 8.29e-08 | 5.78e-07 |
| hsa-miR-30a | -2.16951 | 8.30e-08 | 5.78e-07 |
| hsa-miR-1202 | -2.263009 | 8.48e-08 | 5.87e-07 |
| hsa-miR-1202 | -2.241991 | 8.88e-08 | 6.10e-07 |
| hsa-miR-1202 | -2.22312 | 8.90e-08 | 6.10e-07 |
| hsa-miR-143 | -2.358987 | 9.52e-08 | 6.52e-07 |
| hsa-miR-1202 | -2.233211 | 9.88e-08 | 6.73e-07 |
| hsa-miR-1202 | -2.199345 | 1.18e-07 | 7.85e-07 |
| hsa-miR-654-3p | -2.066709 | 1.19e-07 | 7.93e-07 |
| hsa-miR-335* | -2.233259 | 1.22e-07 | 8.11e-07 |
| hsa-miR-1202 | -2.184557 | 1.34e-07 | 8.83e-07 |
| hsa-miR-1202 | -2.210105 | 1.36e-07 | 8.91e-07 |
| hsa-miR-1202 | -2.173983 | 1.42e-07 | 9.25e-07 |
| hsa-miR-199b-5p | -2.269204 | 1.89e-07 | 1.18e-06 |
| hsa-miR-30a | -2.111923 | 2.08e-07 | 1.28e-06 |
| hsa-miR-133b | -2.065076 | 2.17e-07 | 1.32e-06 |
| hsv1-miR-H1_v14.0 | -2.135519 | 2.36e-07 | 1.40e-06 |
| hsa-miR-199b-5p | -2.442134 | 2.46e-07 | 1.46e-06 |
| hsa-miR-199b-5p | -2.498557 | 2.58e-07 | 1.51e-06 |
| hsa-miR-199b-5p | -2.467045 | 3.01e-07 | 1.71e-06 |
| hsa-miR-199b-5p | -2.40785 | 3.32e-07 | 1.86e-06 |
| hsa-miR-199b-5p | -2.19188 | 3.91e-07 | 2.11e-06 |
| hsa-miR-199b-5p | -2.15159 | 4.58e-07 | 2.41e-06 |
| hsa-miR-199b-5p | -2.349763 | 4.93e-07 | 2.58e-06 |
| hsa-miR-199b-5p | -2.332505 | 4.96e-07 | 2.58e-06 |
| hsa-miR-199b-5p | -2.18338 | 5.31e-07 | 2.74e-06 |
| hsv1-miR-H1_v14.0 | -2.414202 | 5.44e-07 | 2.79e-06 |
| hsa-miR-199b-5p | -2.172624 | 5.98e-07 | 3.03e-06 |
| hsa-miR-199b-5p | -2.187524 | 7.39e-07 | 3.65e-06 |
| hsa-miR-199b-5p | -2.153513 | 7.61e-07 | 3.75e-06 |
| hsa-miR-199b-5p | -2.136821 | 7.63e-07 | 3.76e-06 |
| hsa-miR-199b-5p | -2.371091 | 1.59e-06 | 7.37e-06 |
| hsa-miR-495 | -2.040742 | 2.44e-06 | 1.09e-05 |
| hsv1-miR-H1_v14.0 | -2.072784 | 4.36e-06 | 1.85e-05 |

**Table S3. The predicted upstream regulatory miRNAs of DNMT1 from the mirDIP database**

| Gene Symbol | Uniprot | MicroRNA | Integrated Score |
| --- | --- | --- | --- |
| DNMT1 | P26358 | hsa-miR-152-3p | 0.890186720788521 |
| DNMT1 | P26358 | hsa-miR-148a-3p | 0.870981853193031 |
| DNMT1 | P26358 | hsa-miR-148b-3p | 0.868938255665669 |
| DNMT1 | P26358 | hsa-miR-185-5p | 0.642532047226164 |
| DNMT1 | P26358 | hsa-miR-217 | 0.627762205333256 |
| DNMT1 | P26358 | hsa-miR-548aj-3p | 0.590809030689391 |
| DNMT1 | P26358 | hsa-miR-142-5p | 0.57019415146907 |
| DNMT1 | P26358 | hsa-miR-548x-3p | 0.561410937399909 |
| DNMT1 | P26358 | hsa-miR-548am-3p | 0.554426044367593 |
| DNMT1 | P26358 | hsa-miR-548ah-3p | 0.55376697878191 |
| DNMT1 | P26358 | hsa-miR-1264 | 0.551860787194794 |
| DNMT1 | P26358 | hsa-miR-548k | 0.526241577471408 |
| DNMT1 | P26358 | hsa-miR-548ae-3p | 0.520670857465549 |
| DNMT1 | P26358 | hsa-miR-342-3p | 0.502436726265566 |
| DNMT1 | P26358 | hsa-miR-34c-3p | 0.499964654656194 |
| DNMT1 | P26358 | hsa-miR-3157-5p | 0.484313626734879 |
| DNMT1 | P26358 | hsa-miR-140-5p | 0.47528271689082 |
